# Comorbidities and their association with COVID-19 mortality in Mexico between January 2020 and August 2021

**DOI:** 10.1371/journal.pone.0296895

**Published:** 2024-04-17

**Authors:** Ryan J. Lowhorn, Mohammed Chowdhury, Symon Kimitei, Sammie Haskin, Mohammad Masum, A. K. M. Fazlur Rahman

**Affiliations:** 1 School of Data Science and Analytics, Kennesaw State University, Kennesaw, Georgia, United States of America; 2 College of Business and Technology, Western Illinois University, Macomb, Illinois, United States of America; 3 San Jose State University, San Jose, California, United States of America; 4 Department of Biostatistics, University of Alabama, Birmingham, Alabama, United States of America; Chulalongkorn University Faculty of Medicine, THAILAND

## Abstract

By August 17, 2021, 4.3 million people had died globally as a result of SARS-CoV-2 infection. While data collection is ongoing, it is abundantly obvious that this is one of the most significant public health crises in modern history. Consequently, global efforts are being made to attain a greater understanding of this disease and to identify risk factors associated with more severe outcomes. The goal of this study is to identify clinical characteristics and risk factors associated with COVID-19 mortality in Mexico. The dataset used in this study was released by Sistema Nacional de Vigilancia Epidemiologica de Enfermedades Respiratorias (SISVER) de la Secretaría de Salud and contains 2.9 million COVID-19 cases. The effects of risk factors on COVID-19 mortality were estimated using multivariable logistic regression models with generalized estimation equation and Kaplan-Meier curves. Case fatality rates, case hospitalization rates are also reported using the Centers for Disease Control and Prevention (CDC) USA death-to-case ratio method. In general, older males with pre-existing conditions had higher odds of death. Age greater than 40, male sex, hypertension, diabetes, and obesity are associated with higher COVID-19 mortality. End-stage renal disease, chronic obstructive pulmonary disease, and immunosuppression are all linked with COVID-19 patient fatalities. Smoking and Asthma are associated with lower COVID-19 mortality which is consistent with findings from the article published in Nature based on National Health Service (NHS) of UK dataset (17 million cases). Intensive care unit (ICU), patient intubation, and pneumonia diagnosis are shown to substantially increase mortality risk for COVID-19 patients.

## Introduction

People continue to suffer worldwide due to the sickness emerged from the SARS-CoV-2 virus, dubbed COVID-19. As of August 17, 2021, approximately 207 million instances of COVID-19 had been documented worldwide, with over 4.3 million fatalities [1]. Consequently, the COVID-19 pandemic is now largely regarded as one of the most consequential public health issues in contemporary medicine. While several studies have analyzed the demographic characteristics and comorbidities of COVID-19 patients in various periods and waves, our study aims to build upon this existing knowledge. With the expansion of sample sizes, we anticipate uncovering nuances in data trends not previously observable in smaller datasets. It’s crucial to understand how our findings compare and contrast with previous research, especially as we aim to identify any new insights or validate previously observed patterns. As more data become available, our understanding of COVID-19 infection will improve, and risk factors for serious health effects, such as death, will become more accurate. COVID-19 datasets with patient-level COVID-19 data are few [2]. As a result, conducting a comprehensive large-scale study of patients positive for COVID-19 is challenging but nevertheless essential.

Since January of 2020, Sistema Nacional de Vigilancia Epidemiologica de Enfermedades Respiratorias (SISVER) de la Secretaría de Salud has been collecting data of individuals diagnosed with COVID-19. This dataset consists of clinical and demographic data gathered from clinics and hospitals (public & private) across Mexico. This database has been consistently updated since January 2020 and has been made publicly available through the Secretaría de Salud (Secretariat of Health) (https://www.gob.mx/salud/documentos/datos-abiertos-152127).

Large-scale examination of data demonstrating individual case comorbidities in COVID-19 individuals has been quite restricted in contrast to what is required [2]. As information becomes more readily available, it is critical to do research on the most recent datasets available to ensure the most accurate results, as more recent data may provide different mortality rates, comorbidity percentages, and so on. Based on the patient level data, we demonstrate that this study offers more information on the risks of death associated with various characteristics found in patients positive for COVID-19.

## Materials and methods

### Data sourcing

The COVID-19 database provided by SISVER of Ministry of Health (MoH), Mexico has been monitoring cases since January 1, 2020 and is updated daily. Each patient in the dataset is registered by one of the 475 Viral Repository Disease Monitoring Units spread throughout Mexico in public and private sectors of work. These data sets are published through SISVER or Red Nacional de Laboratorios de Vigilancia Epidemiologica. The original dataset contained 9,156,983 patients. The cases confirmed to have a final classification of COVID-19 positive (through reverse transcription polymerase chain reaction (RT-PCR) or antigen tests) were extracted and cleaned for our analysis by scanning for missing values in the dataset. The dataset contained 18,546 individuals who lacked sufficient data due mainly to missing values for comorbidity variables and hospitalization status. To address this issue, we employed multivariate imputation by Chained equations (MICE) [3] via the MICE package in R [3, 4]. Using MICE, we imputed missing values for each case using the Akaike Information Criterion (AIC). The total number of COVID-19 cases used in this study was 2,909,453. The cases retained in these analyses were laboratory confirmed to be positive for COVID-19 through RT-PCR or antigen tests. We considered both data (with imputation for missing values and without imputation) for analysis.

### Covariates

The variables included from the dataset for this study were demographic (sex and age), medical history (patient comorbidities, smoking habits, patient pregnancy), and epidemiological characteristics such as COVID-19 confirmation, and COVID-19 contact. The laboratory and clinical data included the following: patient intubation, patient hospitalization, hospital admission date, pneumonia confirmation, intensive care unit admission (ICU). The date of death is available, but the recovery date is not recorded in the data set.

In this analysis, all dichotomous variables apart from patient sex, consist of discrete “yes” or “no” values. The “not applicable” value refers to patients who do not fall into either category. For the COVID-19 status section, intubation and ICU were included as polytomous variables labeled “yes,” “no,” or “not applicable.” Hospitalized and pneumonia are also included in the COVID-19 status section and are dichotomous. In the dichotomous comorbidities section, variables are: hypertension, obesity, CVD (cardiovascular disease), diabetes, COPD (chronic obstructive pulmonary disease), asthma, immunosuppression, ESRD (end stage renal disease), and other comorbidities. In the demographics section, a dichotomous sex and continuous age variables are included. Dichotomous variables labelled COVID-19 contact and smoking habit plus a polytomous pregnancy variable with values labelled “yes,” “no,” or “not applicable” are also included in this analysis. A polytomous healthcare variable with thirteen healthcare values (healthcare providers) is also included. The healthcare values can be seen in Table 1.

**Table 1:** Summary of demographics, COVID-19 status, and comorbidity variables of all COVID-19 patients from January 29, 2020–August 16, 2021 in Mexico.

	*Overall (N = 2909453)*	*Alive (N = 2681580)*	*Deceased (N = 227873)*	*CFR % (O = 7.7)*	*Hospitalized (N = 485573)*	*CHR% (O = 16.7)*
*Age Median (Q1, Q3)*	40.0 [29.0, 53.0]	39.0 [28.0, 51.0]	64.0 [54.0, 73.0]		58.0 [46.0, 69.0]	
***Demographics***						
*Age Group*						
*0–17*	156724 (5.4%)	155997 (5.8%)	727 (0.3%)	0.5	7418 (1.5%)	4.7
*18–29*	606032 (20.8%)	603123 (22.5%)	2909 (1.3%)	0.5	20131 (4.1%)	3.3
*30–39*	649296 (22.3%)	639306(23.8%)	9900 (4.4%)	1.5	45111 (9.3%)	6.9
*40–49*	578469 (19.9%)	552862 (20.6%)	25607 (11.2%)	4.4	77331 (15.9%)	13.4
*50–64*	626128 (21.5%)	541559 (20.2%)	84569 (37.1%)	13.5	175883 (36.2%)	28.1
*65–79*	229510 (7.9%)	153491 (5.7%)	76019 (33.4%)	33.1	119848 (24.7%)	52.2
*≥ 80*	63294 (2.2%)	35242 (1.3%)	28052 (12.3%)	44.3	39851 (8.2%)	63.0
*Sex*						
*Female*	1450701 (49.9%)	1364334 (50.9%)	86367 (37.9%)	5.9	199309 (41.0%)	13.9
*Male*	1458752 (50.1%)	1317246 (49.1%)	141506 (62.1%)	9.7	286264 (59.0%)	19.6
***Pregnant***						
*Not Applicable*	1458752 (50.1%)	1317246 (49.1%)	141506 (62.1%)	9.7	286264 (59.0%)	19.6
*No*	1430730 (49.2%)	1344587 (50.1%)	86143 (37.8%)	6.0	195451 (40.3%)	13.7
*Yes*	19971 (0.7%)	19747 (0.7%)	224 (0.1%)	1.1	3858 (0.8%)	19.3
***Smoking habit***						
*No*	2708072 (93.1%)	2497541 (93.1%)	210531 (92.4%)	7.8	449877 (92.6%)	16.6
*Yes*	201381 (6.9%)	184039 (6.9%)	17342 (7.6%)	8.6	35696 (7.4%)	17.1
***COVID-19 contact***						
*No*	1705763 (58.6%)	1528915 (57.0%)	176848 (77.6%)	10.4	364625 (75.1%)	21.2
*Yes*	1203690 (41.4%)	1152665 (43.0%)	51025 (22.4%)	4.2	120948 (24.9%)	9.9
***COVID-19 status***						
*Deceased*						
*No*					276032 (56.8%)	10.2
*Yes*					209541 (43.2%)	91.9
*Hospitalized*						
*No*	2423880 (83.3%)	2405548 (89.7%)	18332 (8.0%)	0.8	-------	-------
*Yes*	485573 (16.7%)	276032 (10.3%)	209541 (92.0%)	43.2	-------	-------
*ICU*						
*Not Applicable*	2423880 (83.3%)	2405548 (89.7%)	18332 (8.0%)	0.8	-------	-------
*No*	447106 (15.4%)	258916 (9.7%)	188190 (82.6%)	42.1	447106 (92.1%)	100
*Yes*	38467 (1.3%)	17116 (0.6%)	21351 (9.4%)	55.5	38467 (7.9%)	100
*Intubated*						
*Not Applicable*	2423880 (83.3%)	2405548 (89.7%)	18332 (8.0%)	0.8		
*No*	423944 (14.6%)	265112 (9.9%)	158832 (69.7%)	37.5	423944 (87.3%)	100
*Yes*	61629 (2.1%)	10920 (0.4%)	50709 (22.3%)	82.3	61629 (12.7%)	100
*Pneumonia*						
*No*	2544708 (87.5%)	2480716 (92.5%)	63992 (28.1%)	2.5	171432 (35.3%)	6.7
*Yes*	364745 (12.5%)	200864 (7.5%)	163881 (71.9%)	44.9	314141 (64.7%)	86.1
***Comorbidities***						
*Diabetes*						
*No*	2563441 (88.1%)	2420155 (90.3%)	143286 (62.9%)	5.6	332050 (68.4%)	13.0
*Yes*	346012 (11.9%)	261425 (9.7%)	84587 (37.1%)	24.4	153523 (31.6%)	44.4
*COPD*						
*No*	2881338 (99.0%)	2663395 (99.3%)	217943 (95.6%)	7.6	469338 (96.7%)	16.3
*Yes*	28115 (1.0%)	18185 (0.7%)	9930 (4.4%)	35.3	16235 (3.3%)	57.7
*Immunosuppression*						
*No*	2888324 (99.3%)	2665495 (99.4%)	222829 (97.8%)	7.7	475857 (98.0%)	16.5
*Yes*	21129 (0.7%)	16085 (0.6%)	5044 (2.2%)	23.9	9716 (2.0%)	45.6
*Hypertension*						
*No*	2459603 (84.5%)	2334296 (87.0%)	125307 (55.0%)	5.1	304573 (62.7%)	12.4
*Yes*	449850 (15.5%)	347284 (13.0%)	102566 (45.0%)	22.8	181000 (37.3%)	40.2
*Other comorbidity*						
*No*	2856813 (98.2%)	2640372 (98.5%)	216441 (95.0%)	7.6	463489 (95.5%)	16.2
*Yes*	52640 (1.8%)	41208 (1.5%)	11432 (5.0%)	21.7	22084 (4.5%)	42.0
*CVD*						
*No*	2870137 (98.6%)	2653619 (99.0%)	216518 (95.0%)	7.5	465965 (96.0%)	16.2
*Yes*	39316 (1.4%)	27961 (1.0%)	11355 (5.0%)	28.9	19608 (4.0%)	49.9
*Obesity*						
*No*	2521360 (86.7%)	2344692 (87.4%)	176668 (77.5%)	7.0	382450 (78.8%)	15.2
*Yes*	388093 (13.3%)	336888 (12.6%)	51205 (22.5%)	13.2	103123 (21.2%)	26.6
*ESRD*						
*No*	2871247 (98.7%)	2658743 (99.1%)	212504 (93.3%)	7.4	460862 (94.9%)	16.1
*Yes*	38206 (1.3%)	22837 (0.9%)	15369 (6.7%)	40.2	24711 (5.1%)	64.7
*Asthma*						
*No*	2848766 (97.9%)	2624986 (97.9%)	223780 (98.2%)	7.9	475718 (98.0%)	16.7
*Yes*	60687 (2.1%)	56594 (2.1%)	4093 (1.8%)	6.7	9855 (2.0%)	16.2
***Healthcare***						
*Red Cross*	300 (0.0%)	275 (0.0%)	25 (0.0%)	8.3	32 (0.0%)	10.7
*DIF*	829 (0.0%)	827 (0.0%)	2 (0.0%)	0.2	29 (0.0%)	3.5
*State*	31989 (1.1%)	28424 (1.1%)	3565 (1.6%)	11.1	7928 (1.6%)	24.8
*IMSS*	1107704 (38.1%)	977558 (36.5%)	130146 (57.1%)	11.7	250501 (51.6%)	22.6
*IMSS-Welfare*	18752 (0.6%)	16884 (0.6%)	1868 (0.8%)	10.0	4600 (0.9%)	24.5
*ISSSTE*	92458 (3.2%)	75901 (2.8%)	16557 (7.3%)	17.9	35898 (7.4%)	38.8
*Municipal*	1816 (0.1%)	1733 (0.1%)	83 (0.0%)	4.6	985 (0.2%)	54.2
*State oil*	18088 (0.6%)	15082 (0.6%)	3006 (1.3%)	16.6	7731 (1.6%)	42.7
*Private*	79698 (2.7%)	75765 (2.8%)	3933 (1.7%)	4.9	16674 (3.4%)	20.9
*Armed forces*	14033 (0.5%)	12122 (0.5%)	1911 (0.8%)	13.6	7850 (1.6%)	55.9
*Navy*	9408 (0.3%)	8569 (0.3%)	839 (0.4%)	8.9	2511 (0.5%)	26.7
*SSA*	1532266 (52.7%)	1466766 (54.7%)	65500 (28.7%)	4.3	149870 (30.9%)	9.8
*Universal*	2112 (0.1%)	1674 (0.1%)	438 (0.2%)	20.7	964 (0.2%)	45.6

Comorbidity percentages are not mutually disjoint. The fatality rate was estimated using the CDC death-to-case ratio method. CVD = cardiovascular disease; COPD = chronic obstructive pulmonary disease; ESRD = end state renal disease; CHR = Case Hospitalization Rate; CFR = Case Fatality Rate = (number of deaths of a specific condition. / total number cases with that specific condition) *100 per 100 case.

The primary outcome variable used in this study is confirmation of death (Yes/No) from COVID positive patients. Patients whose death were not reported by the end of the study period August 16, 2021, were considered alive. In addition, case fatality rates (CFR), case hospitalization rates (CHR) are also reported using the Center for Disease Control (CDC) death-to-case ratio method [5]. For example, for age ≥ 80 years,

$$CFR\%=\frac{number\;of\;COVID\;related\;deaths\;of\;age\;\geq \;80\;years\;}{total\;COVID\;positive\;cases\;of\;age\;\geq \;80\;years}*100$$

### Statistical analysis

All analyses were done using the R programming language [4] (version 4.1.0). Descriptive analyses [mean (standard deviation), median (interquartile range), and frequency (%) as appropriate] were used to describe study cohort stratified by mortality status. Case fatality rates, case hospitalization rates are also reported using the CDC death-to-case ratio method. Chi-square or Wilcoxon rank-sum tests were performed as appropriate to compare clinical characteristics and comorbidities between groups.

Multivariable logistic regression models were performed to identify the risk factors associated with COVID-19 mortality. Adjusted odds ratios (aOR) with 95% confidence interval (CI) were estimated to quantify the effects of risk factors on the COVID-19 mortality. To estimate more statistically robust odds ratios, a generalized estimation equations (GEE) models were employed which used healthcare provider as the clustering variable. The covariates included in this model are: sex, age, confirmed pneumonia, diabetes, COPD, CVD, asthma, ESRD, Immunosuppression, smoking habits, hypertension, and obesity. In this analysis, age was grouped into the following subgroups: 0–17, 18–29, 30–39, 40–49, 50–64, 65–79, and ≥80 years. A second multivariable logistic regression model with GEE was also performed for hospitalized patients including two additional covariates ICU and intubation status in the model. Variables for these two models were selected through use of the Akaike Information Criterion (AIC).

Kaplan-Meier survival probability plots were constructed for COVID-19 patients using the survminer [6] and ggplot2 package [7] for eight covariates. These plots are constructed to demonstrate the survival probability of male and female patients with different demographics, comorbidity conditions and patient status. The covariates used in the Kaplan-Meier survival curves are pneumonia, diabetes, COPD, ESRD, Immunosuppression, other comorbidities, hypertension, and obesity. Log-rank tests were performed to compare survival curves between groups. A sensitivity analysis was performed with complete observations (not replacing missing values using imputation methods).

## Results

### Demographics

This analysis contained 2,909,453 COVID-19 patients. Of all patients positive with COVID-19, 41.4% had contact history with other COVID-19 patients. The median age of the individuals studied was 40.0 [29.0, 53.0] years and 49.9% of the patients were female. Patients’ characteristics stratified by mortality status are summarized in Table 1. Positive cases were shown to be most prevalent in the age group 30–39 (22.3%) and least prevalent in the age group ≥80 (2.2%). Fatality rates were highest among those belonging to the two oldest age groups ≥80 (44.3%) and 65–79 (33.1%) respectively (Table 1). The cumulative fatality rate for COVID-19 patients is shown to be approximately 7.7%. Patient deaths were most concentrated in the age group 50–64 (37.1%) followed by age group 65–79 (33.4%) and the lowest in the age group 0–17 (0.3%). The median age of patients who did not survive was 64.0 [54.0, 73.0] years and those who survived was 39.0 [28.0, 51.0]. The median age of male and female COVID-19 patients who had died were 63 and 65 years respectively. Pregnant patients were shown to have a low fatality rate of 1.1%.

**Fig 1:**
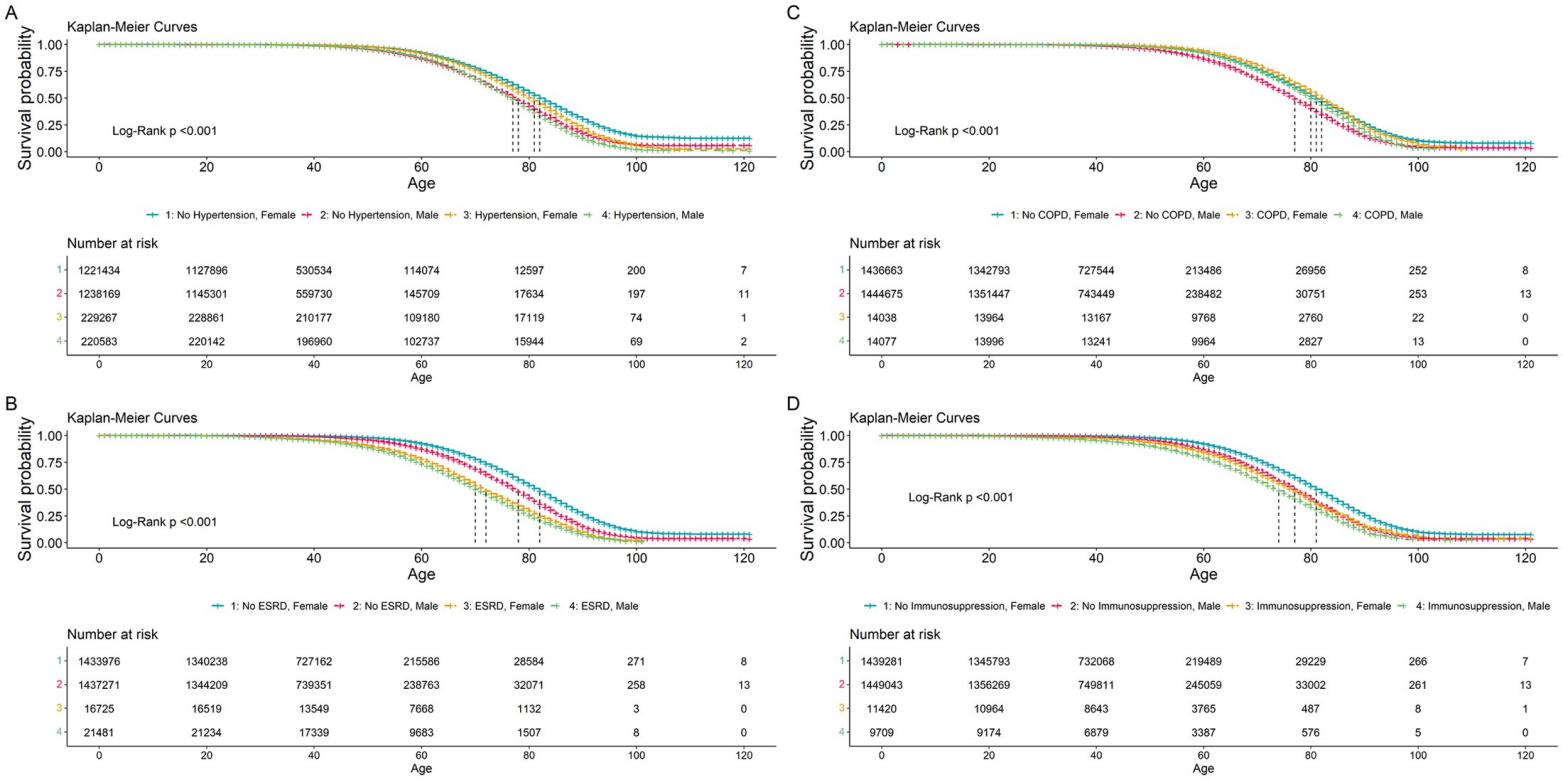
All Mexico COVID-19 patients from January 29, 2020 –August 16, 2021. Kaplan-Meier survival probability plots for male and female COVID-19 patients consisting of the following characteristics: with and without (A) hypertension, (B) ESRD (end stage renal disease), (C) COPD (chronic obstructive pulmonary disease), and (D) Immunosuppression. Adjacent vertical lines in each figure indicates which two curves are compared.

**Fig 2:**
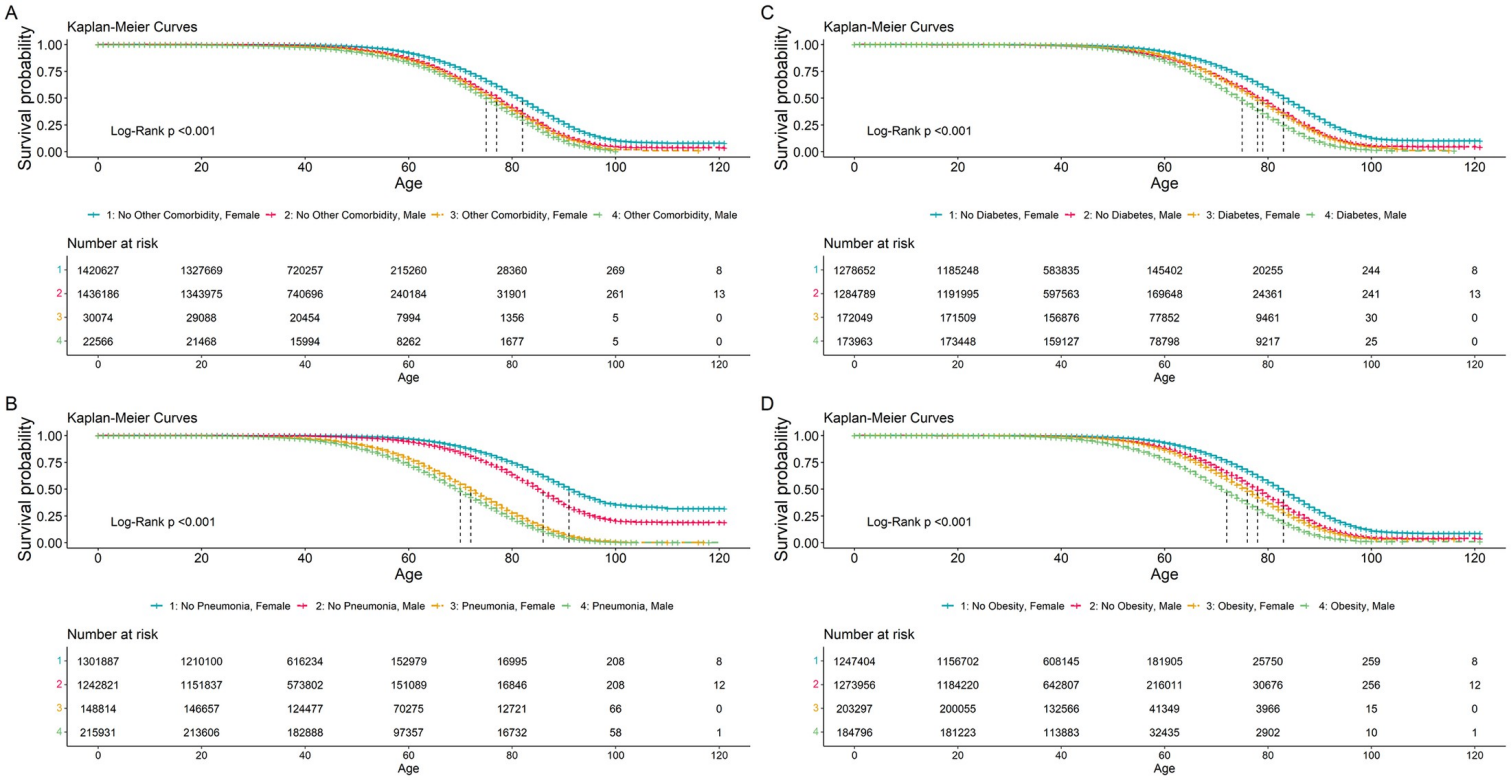
All Mexico COVID-19 patients from January 29, 2020 –August 16, 2021. Kaplan-Meier survival probability plots for male and female COVID-19 patients consisting of the following characteristics: with and without (A) other comorbidity), (B) pneumonia (C) diabetes, and (D) obesity. Adjacent vertical lines in each figure indicates which two curves are compared.

To estimate odds ratios (OR) for the different covariates, multivariable logistic regression model with a GEE is fitted for all positive COVID-19 patients (Tables 2 and 3). Starting with the age variable, the adjusted odds ratios (aOR) show that as age increases, the odds of death increase (P < 0.001). Specifically, compared to the reference age group 30–39, odds of death were greater for age groups 40–49, 50–64, 65–79, and ≥80 with aORs and 95% CIs (confidence interval) were 2.15 (95% CI 2.10–2.20), 4.89 (95% CI 4.78–5.00), 11.58 (95% CI 11.31–11.86), and 18.08 (95% CI 17.56–18.62), respectively. Of the comparison to the 30–39 reference age group, odds of death were less for age groups 0–17 (aOR = 0.45, 95% CI 0.42–0.49) and 18–29 (aOR = 0.43, 95% CI, 0.4–0.45). The higher aOR among older age groups indicated that people in older age groups had disproportionally higher odds of death due to COVID-19 regardless of patient sex. Male patients had 1.61 (95% CI 1.59–1.63) times higher odds of death compared to female patients. Kaplan-Meier curves demonstrated that male patients had a substantially increased risk of mortality when compared to female patients. Sharp survival declines can be seen in the survival plot curves as age increased beyond the mid-range age group of 40–50 years (Figs 1 and 2).

**Table 2:** Adjusted odds ratios with 95% confidence interval from multivariable logistic regression model with GEE for the outcome COVID-19 mortality.

Predictors	Adjusted Odds Ratios	CI 95%	Wald Statisitc	P-value
**Demographics**				
Age Group				
30–39 (Ref)	1.0			
0–17	0.45	0.42–0.49	526.09	**<0.001**
18–29	0.43	0.41–0.45	1847.83	**<0.001**
40–49	2.15	2.10–2.20	4128.11	**<0.001**
50–64	4.89	4.78–5.00	20573.17	**<0.001**
65–79	11.58	11.31–11.86	41204.29	**<0.001**
≥80	18.08	17.56–18.62	35136.56	**<0.001**
**Sex**				
Female	1.0			
Male	1.61	1.59–1.63	6803.51	**<0.001**
**Smoker**				
No	1.0			
Yes	0.9	0.88–0.92	81.19	**<0.001**
**COVID-19 status**				
Pneumonia				
No	1.0			
Yes	14.51	14.35–14.67	203274.6	**<0.001**
**Comorbidities**				
No comorbidity	1.0			
Diabetes	1.42	1.40–1.44	2548.16	**<0.001**
COPD	1.27	1.23–1.31	153.36	**<0.001**
Immunosuppression	1.45	1.39–1.52	218.97	**<0.001**
Hypertension	1.31	1.29–1.32	1565.74	**<0.001**
Other comorbidity	1.74	1.69–1.79	1043.10	**<0.001**
CVD	1.02	0.99–1.05	1.61	0.15
Obesity	1.4	1.38–1.42	2111.28	**<0.001**
ESRD	2.72	2.65–2.80	3277.77	**<0.001**
Asthma	0.89	0.85–0.92	31.29	**<0.001**

The analysis includes n = 2,909,453 observation (all Mexico COVID-19 positive patients from January 29, 2020–August 16, 2021); aOR = adjusted odds ratio; CI = confidence interval; P = P-value; COPD = chronic obstructive pulmonary disease; CVD = cardiovascular disease; ESRD = end stage renal disease.

**Table 3:** Adjusted odds ratios with 95% confidence interval from multivariable logistic regression model with GEE for the outcome COVID-19 mortality for only hospitalized patients.

Predictors	Adjusted Odds Ratios	CI 95%	P-value
**Demographics**			
Age Group			
30–39 (Ref)	1.0		
0–17,	0.44	0.40–0.48	**<0.001**
18–29	0.66	0.63–0.70	**<0.001**
40–49	1.63	1.58–1.68	**<0.001**
50–64	2.88	2.80–2.96	**<0.001**
65–79	5.28	5.14–5.44	**<0.001**
≥80	7.56	7.31–7.82	**<0.001**
**Sex**			
Female	1.0		
Male	1.3	1.28–1.31	**<0.001**
**Smoking Habit**			
No	1.0		
Yes	0.9	0.88–0.92	**<0.001**
**COVID-19 status**			
ICU			
No	1.0		
Yes	1.41	1.37–1.45	**<0.001**
Intubated			
No	1.0		
Yes	8.58	8.37–8.79	**<0.001**
Pneumonia			
No	1.0		
Yes	1.66	1.63–1.68	**<0.001**
**Comorbidities**			
No comorbidity	1.0		
Diabetes	1.14	1.13–1.16	**<0.001**
COPD	1.08	1.04–1.12	**<0.001**
Immunosuppression	1.2	1.15–1.26	**<0.001**
Hypertension	1.16	1.14–1.18	**<0.001**
Other comorbidity	1.19	1.16–1.23	**<0.001**
CVD	0.94	0.91–0.97	**<0.001**
Obesity	1.14	1.13–1.16	**<0.001**
ESRD	1.76	1.70–1.81	**<0.001**
Asthma	0.88	0.84–0.92	**<0.001**

The analysis includes n = 485,573 observation (all COVID-19 positive hospitalized patients (from January 29, 2020–August 16, 2021); aOR = adjusted odds ratio; CI = confidence interval; P = P-value; COPD = chronic obstructive pulmonary disease; CVD = cardiovascular disease; ESRD = end stage renal disease; ICU = intensive care unit.

### COVID-19 patient status

One in every six (16.7%) COVID-19 patients were hospitalized. Hospitalized patients had a fatality rate of 43.2%, whereas non-hospitalized patients had a fatality rate of approximately 0.8%. Approximately 12.5% of patients were diagnosed with pneumonia. Patients with pneumonia diagnoses had higher fatality rate and higher hospitalization rate: 43.2% and 86.1% respectively, compared to patients with no pneumonia diagnoses, 2.5% and 6.7% respectively. Intubated patients were shown to have a fatality rate of 82.3%, comprising 2.1% of COVID-19 patients. ICU admitted patients had a fatality rate of 55.5% and comprised 1.3% of COVID-19 patients. On the multivariable logistic regression model with GEE for the whole cohort (both hospitalized and non-hospitalized patients), pneumonia is shown to be significantly (OR = 14.51, 95% CI: 14.4–14.67, P < 0.001) associated with increased mortality. From the Kaplan-Meier estimates, it is shown that Females with no comorbidities have the highest survival probability (Figs 1 and 2). In all Kaplan-Meier estimates, male patients are shown to have lower survival probabilities when compared to female patients. Interestingly, survival probability was significantly affected by pneumonia regardless of patient sex (Fig 2(B)). As age increases beyond 40 years, survival probability sharply decreases regardless of patient sex (Figs 1 and 2).

### Comorbidities

The three most prevalent comorbidities of positive COVID-19 patients were hypertension, obesity, and diabetes with percentages of 15.5%, 13.3%, and 11.9% respectively. Moreover, the fatality rates of hypertension, obesity, and diabetes were 22.8%, 13.2%, and 24.4% respectively (Table 1). Looking at the largest comorbidities in this study, diabetes, obesity, and hypertension had the highest odds of death with adjusted odd ratios of 1.42 (95% CI 1.40–1.44), 1.40 (95% CI 1.38–1.42), and 1.31 (95% CI 1.29–1.32) respectively. All comorbidities (Table 1) were shown to be significantly related to mortality in COVID-19 patients (P < 0.001) except cardiovascular disease (Table 2).

The less frequent comorbidities: ESRD (1.3%; aOR = 2.72, 95% CI: 2.65–2.80), other comorbidities (1.8%; aOR = 1.74, 95% CI: 1.69–1.79), immunosuppressive (0.7%; aOR = 1.45, 95% CI: 1.39–1.52), COPD (1.0%, aOR = 1.27, 95% CI: 1.23–1.31), and CVD (1.4%; aOR = 1.02, 95% CI: 0.99–1.05), all were associated with increased COVID-19 mortality. Surprisingly the other low frequent (2.1%) comorbidity Asthma was associated with decreased (OR = 0.89, 95% CI: 0.85–0.92) COVID-19 mortality.

Patients with smoking habits had a fatality rate of 8.6% while those who did not, had a fatality rate of 7.8%. On the adjusted logistic regression model, smoking is associated with lower (aOR = 0.90, 95% CI:.88–0.92) COVID-19 mortality. This result is consistent with results reported in the articles [8] on UK NHS hospital data with approximately 17 million cases and another data from France [9].

On the multivariable logistic regression with a GEE model for only hospitalized patients, intubated patients are shown to have much higher odds of mortality than non-intubated patients with an aOR of 8.58 (95% CI 8.37–8.79) (Table 3). ICU is shown to be associated with 29% reduction in mortally (aOR = 0.71, 95% CI: 0.69–0.73, P <0.001). For hospitalized patients, pneumonia is associated with higher (aOR = 1.66) COVID-19 mortality whereas for the whole cohort, pneumonia is associated much higher (aOR = 14.51) COVID-19 mortality. CVD is associated with lower COVID-19 mortality for hospitalized patients in contrast to association with mortality when included in the whole cohort model. The effects of all other factors on COVID-19 mortality remain similar in both models.

## Discussion

To our knowledge, at the time of our study, this represented one of the most extensive databases examining COVID-19 patients in Mexico, analyzing both demographic characteristics and comorbidities. It also stands as one of the largest such datasets on a global scale. Our results indicate that male patients had higher risk of death. Specifically, males middle-aged (≥ 40 years) with pre-existing conditions (e.g., diabetes, obesity, and hypertension) had highest odds of death due to COVID-19. This finding is consistent with a separate COVID-19 study of the Mexican population in 2020, which had significantly smaller scale data [10] than this study. Another similar result was reported on the Chinese population in 2020 [11] though specific variations were found in their studies (e.g., p-values and odds ratios). In our study, age showed strong impact on odds of death in both male and female patients positive for COVID-19. Patients with age group 50 to 64 years were shown to have a mortality odds ratio nearly five times that of the reference age group (30–39) and more than 11 times higher for the age group 65 to 79 years. This finding provides further support for studies that found age to be strongly associated with the risk of death in COVID-19 patients [12]

The male and female sex had nearly equivalent total infections with the male sex being slightly greater by 0.2%. These results indicate increased male to female case occurrences when compared to a small-scale COVID-19 study on the Mexican population in 2020, which documented infection percentages of 46.2% and 53.8% in the male and female population respectively. Interestingly, in male and female COVID-19 patients who have died, we observed that the median age of female was 2 years greater than male (female: 65, IQR: 55–74 vs male: 63, IQR: 53–73). This observation further shows that age is a much larger factor than sex in terms of mortality risk.

Smoking habits were confirmed in about 6.9% of cases and CFR is slightly higher for smoker (8.6%) than non-smoker (7.8%). However, on the multivariable logistic regression, smoking is associated with lower risk of COVID-19 mortality (aOR = 0.9, 95% CI: 0.88–0.92). This may seem a counterintuitive result, however, this is not reported first time in the literature. Based on 17 million COVID-19 cases from UK National Health Service (NHS) hospital data, an article published in Nature reported that smoking status is associated with lower COVID-19 mortality [8]. A similar association is also reported in an article [9] based on data from France. Some other studies reported that smoking habits were not shown to be associated with higher mortality in patients with COVID-19 [10, 13]. However, based on relatively much smaller datasets, there are reports that suggest a positive association between smoking and COVID-19 mortality (A = 12–20). There are inconclusive results regarding positive/negative association between smoking status and COVID-19 mortality and may need further study to confirm this association in either direction.

In our study, pneumonia was shown to have very high significance and a high fatality rate of about forty-five percent. The high fatality rate could be attributed to the lung infection by both pneumonia and COVID-19. Cases of COVID-19 with pneumonia are known to commonly require hospitalization [14]. Our analysis also agrees with this as pneumonia had a hospitalization rate of 86.1%. We believe these findings also support the idea that pneumonia is the largest cause for the high fatality rate of patients hospitalized for COVID-19. Hospitalization, ICU admission, and patient intubation were shown to be significantly associated with higher mortality in patients hospitalized for COVID-19. Notably, based on observations in Mexico, a significant proportion of patients who were intubated were not admitted to an ICU, a trend that differs from many other healthcare systems [15]. This observation underscores the importance of infrastructure and specialized care in the management of critical COVID-19 patients in Mexico.

Throughout this study, the most common comorbidities observed in patients were hypertension, obesity, and diabetes respectively. Of the three most prevalent comorbidities, diabetes has been shown to have the highest mortality risk. Diabetes was thought to be heavily associated with disease severity in the times of the Middle East Respiratory Syndrome coronavirus (MERS-CoV) outbreak [14, 16]. Intriguingly, blood glucose levels have been shown to be a case severity predictor in both SARS and COVID-19 cases [17, 18]. This relationship lends additional credence to our observation of an elevated mortality rate in COVID-19 positive individuals afflicted with diabetes. Our finding of obesity being one of the most serious comorbidities for positive COVID-19 patients falls in line with previous research that has shown obesity to heavily aggravate the severity level of COVID-19 [19]. Interestingly, a study in the USA showed obesity as the second most common comorbidity characteristic (41.7%) next to hypertension (56.6%) in patients positive for COVID-19 [20].

In this study, hypertension followed closely behind obesity in terms of risk ratio. Hypertension has been thought to increase the risk of death in patients with COVID-19. A study from China [21] reported that about 58% of COVID-19 patients who needed urgent and intensive care were diagnosed with hypertension while 21.6% of patients who did not require ICU treatment were diagnosed with hypertension. However, this could also be due to the age increase since hypertension tends to be more common in older age groups [22].

The following comorbidities that occur less frequently in our study (immunosuppression, COPD, ESRD) have also been linked to an increased risk of severe infection and death. ESRD has been demonstrated to have the greatest risk of mortality among the less common comorbidities. Additionally, a recent study on ESRD in COVID-19 patients found that the illness had a substantial impact on patient outcome [23]. COVID-19 patients with COPD were shown to have a significantly increased risk of complications and death. A study done in 2020 also showed findings that COVID-19 patients diagnosed with COPD had a larger risk of mortality and severe infection than those who did not have COPD [24]. In that study [24], the researchers explained that SARS-CoV-2 angiotensin-converting-enzyme-2 (ACE2) expression was higher in patients diagnosed with COPD than those who were not. This certainly could explain the increased risk of infection and mortality of patients found in our study but actual tests for increased expression of ACE2 would have to be run to prove a significant relationship. Asthma was also considered to be significantly associated with reduced infection and mortality in patients positive for COVID-19. Interestingly, a 2020 study in Mexico composed of 331,298 patients, found asthma not to be significant (P > 0.01) [25]. This was more than likely due to a smaller analysis of patients. A study done comparing infection and mortality risk in COPD and asthma patients [26] found that patients diagnosed with COPD had a greater risk of developing severe sickness (aOR: 23.433; 95% CI 1.525–360.135; P < 0.01) in comparison to COVID-19 patients diagnosed with asthma, but this could also be linked to the increased ACE2 expression found in COPD patients versus asthma patients. CVD was not shown to be significantly associated with COVID-19 mortality in both hospitalized and not hospitalized patients. Parra-Bracamonte et al. conducted a separate study on the Mexican population in 2020 [10] and found CVD to have an odds ratio less than one (aOR = 0.976, 95% CI: 0.894–1.064) and a non-significant p-value. Moreover, in a COVID-19 study on the Mexican population in 2020, Hernández et al. discovered that CVD was associated with an odds ratio less than one (aOR = 0.93, 95% CI 0.87–1.00) for COVID-19 mortality [26].

It is necessary to be aware of this study’s limitations. There is a lack of specific data on comorbidity features, hospitalization, and cause of death. The severity of comorbidity characteristics at the time of hospitalization or COVID-19 diagnosis might be beneficial in developing a more precise model. The availability of the date of recovery would also have aided us in developing a Cox proportional-hazards model for estimating hospitalization durations in patients with various comorbidities. Additional information on the cause of death may also be beneficial when attempting to develop the most accurate model. Certain factors in the data set, such as city of residence, may be useful in developing a more accurate model if this information is provided for a larger number of patients.

## Conclusions

This study evaluated 2,909,453 patients and collected a wealth of data on the many characteristics associated with patients who tested positive for COVID-19. Our findings indicate that hypertension, followed by obesity and diabetes, are the most often occurring comorbidities in COVID-19 patients and are also some of the comorbidities most strongly related with high mortality rates in the Mexican population. We discovered that individuals over the age of 40 had an increased risk of hospitalization and fatal COVID-19 infection. Smoking was associated with lower risk of COVID-19 mortality. Additionally, COVID-19 patients with ESRD were shown to be at a significantly increased risk of hospitalization and death. Asthma was found to be significant for reduced mortality in patients diagnosed with COVID-19. Patient intubation, ICU admission, and pneumonia are confirmed to be significantly associated with mortality and disease severity in COVID-19 patients. However, more clarity on the pneumonia type and stage may help to further clarify the significance of pneumonia in COVID-19 patients who have died. Patients identified with illnesses that have been demonstrated to enhance the risk of severe illness when exposed to COVID-19 should adhere to local COVID-19 guidelines.
